# *Technical note*: A nonparametric outlier rejection scheme

**DOI:** 10.1155/S1463924692000051

**Published:** 1992

**Authors:** J. S. O. Odonde

**Affiliations:** P.O. Box 151 Terneuzen 4530 AD The Netherlands

## Abstract

Experimental data always contains measurement errors (or noise, in signal processing). This paper is concerned with the removal of outliers from a data set consisting of only a handful of points. The data set has a unimodal probability distribution function, the mode is thus a reliable estimate of the central tendency. The approach is nonparametric; for the data set (x_i_, y_i_) only the ordinates (y_i_) are used. The abscissa (x_i_) are reparametrized to the variable i = 1, N.

The data is bounded using a calculated mode and a new measure: the mean absolute deviation from the mode. This does not seem to have been reported before. The mean is removed and low frequency filtering is performed in the frequency domain, after which the mean is reintroduced.


Journal of Automatic Chemistry, Vol. 14, No. 1 (January–February 1992), pp. 25–27

*Technical note*

### A nonparametric outlier rejection scheme

**J. S. O. Odonde**
*P.O. Box 151, 4530 AD Terneuzen, The Netherlands*

*Experimental data always contains measurement errors (or noise, in signal processing). This paper is concerned with the removal of outliers from a data set consisting of only a handful of points. The data set has a unimodal probability distribution function, the mode is thus a reliable estimate of the central tendency. The approach is nonparametric; for the data set ($x_i$, $y_i$) only the ordinates ($y_i$) are used. The abscissa ($x_i$) are reparametrized to the variable i = 1, N.*

*The data is bounded using a calculated mode and a new measure: the mean absolute deviation from the mode. This does not seem to have been reported before. The mean is removed and low frequency filtering is performed in the frequency domain, after which the mean is reintroduced.*

### Introduction

Consider an experiment where measurements are logged for further calculations. The intention is to use the data points that are most reliable; that is, when the experiment and the measurement device has settled. Two main causes of invalid data (i.e. outliers) are:

(1) The influence of previous experiments.
(2) The effect of the environment (for example mains flicker).

A common and simple practice is to ensure a long experimental run to produce *enough* valid data. If the run length is very large and the measurement errors causing bad data are independent, and normally distributed with a constant standard deviation, the ubiquitous least-squares fit is a *maximum likelihood* estimator of the line parameters. The *weighted* least-squares (or chi-squared) fit relaxes the assumption of a fixed standard deviation.

It is often assumed that a random variable is normally distributed. The *central limit theorem* (see standard text on mathematical statistics and also the references) justifies approximate normality for large sets of data, but it may be difficult to construct a normal distribution from experimental data (Press *et al.* [1] provides good treatment of this problem). Various outlier rejection schemes are based on this normality (for example see *Matlab Users' Guide* [2], Section 5.3).

### Outliers in a small dataset

This paper discusses small datasets with a maximum of 11 members per set. The data is grouped around the true value; the outliers are readings from previous and subsequent experiments. The distribution therefore peaks around the true value.

The data have a *central tendency* towards the true value. Now, the central tendency is characterized by a scalar which is a function of the *moments* of the dataset.

The *mean* is the most commonly used measure of the central tendency. The *variance* is then used to measure the spread about the central tendency.

The *average deviation* or *mean absolution deviation* for the data set $x_i$, i = 1, N is

$$\sigma = \frac{1}{N}\sum_{j=1}^{N}\left|x_j - \bar{x}\right| \qquad (1)$$

where

$\bar{x}$ is the mean.

The mean absolute deviation is recognized as a more robust estimate of the width around the central tendency if the second moment can not be realized (i.e. if it is infinite) [1].

The mean in a small dataset is not representative of the central tendency; the distribution has broad tails. In this case, the *median* (for a probability distribution function p[x], the median $x_{med}$ is the value for which larger and smaller values are equally probable) and the *mode* (for a probability distribution function p[x], the mode $x_{mode}$ is the value at which p[x] is a maximum) are alternative estimations of the true value.

### Mean absolute deviation from the mode

If the median is not representative of the set (which is the case when the area in the tails of the distribution is large; the mean fails if the first moment of the tails is large). Then the mode of the distribution must be evaluated. Subsequent statistics of the set should by necessity, therefore, not involve the mean and the median.

The 'width' about the central tendency can be taken as the mean absolute deviation from the mode:

$$\sigma = \frac{1}{N}\sum_{j=1}^{N}\left|x_j - x_{mode}\right| \qquad (2)$$

the outlier rejection algorithm is then as follows.

### Outlier rejection algorithm

(1) Get $x_{mode}$.

(2) Bounding if

$|x_i - x_{mode}| > \Delta$

then $x_i = x_{mode}$ for i = 1, N.

0142–0453/92 $3.00 © 1992 Taylor & Francis Ltd.

(3) Get mean $\bar{x}$ of bounded data set.

(4) Detrend data

$$s_i = x_i - \bar{x}$$

(5) Take Fast Fourier Transform of set $s_i$.

(6) Low pass filter (multiply by a smooth window function).

(7) Inverse Fast Fourier Transform to give set $r_i$.

(8) Restore mean

$$v_i = r_i - \bar{x}$$

to give the set $v_i$ which has no outliers.

In step 2 above, the criterion $\Delta$ is evaluated as

$$\Delta = \sigma' \rho \quad (3)$$

where

$\sigma'$ is the mean absolute deviation from the mode, and,

$\rho$ is the (maximum) proportion of outlier points. In the examples below, $\rho = 0{\cdot}3$.

A bounded datapoint is set to $x_i = x_{mode}$. In general,

$$X_{i\ bounded} = x_i + \varepsilon_i$$

where a possible

$$\varepsilon_i = -(x_i + x_{mode})\ \theta$$

and

$\theta$ is the porportional contribution of the point $x_i$ to the broad tail of the distribution.

Steps 5 to 7 are executed admirably by the routine SMOOFT in Press[1]. The sequel to the routine suggests that, for distributions with broad tails, a moving window median on all ordinates should be performed. The outlier

*Table 1. Data set 1.*

| Data point | Raw data | Outlier rejected data |
|---|---|---|
| 1 | 3·18 | 0·086 |
| 2 | 0·25 | 0·087 |
| 3 | 0·11 | 0·086 |
| 4 | 0·10 | 0·087 |
| 5 | 0·08 | 0·086 |
| 6 | 0·08 | 0·087 |
| 7 | 0·06 | 0·087 |
| 8 | 0·07 | 0·087 |
| 9 | 0·05 | 0·087 |
| 10 | 10·86 | 0·087 |
| 11 | 10·86 | 0·088 |

*Statistics*

| | Mean | ρ | Standard deviation | Median | ρ' | Mode |
|---|---|---|---|---|---|---|
| Raw | 2·34 | 3·25 | 4·31 | 0·10 | 2·27 | 0·07 |
| Outlier rejected | 0·09 | 0·03 | 0·06 | 0·07 | 0·03 | 0·07 |

*Table 2. Data set 2.*

| Data point | Raw data | Outlier rejected data |
|---|---|---|
| 1 | 76·97 | 43·39 |
| 2 | 77·48 | 43·39 |
| 3 | 47·45 | 43·39 |
| 4 | 46·66 | 43·39 |
| 5 | 44·47 | 43·39 |
| 6 | 44·25 | 43·39 |
| 7 | 43·59 | 43·39 |
| 8 | 43·06 | 43·39 |
| 9 | 42·89 | 43·39 |
| 10 | 42·91 | 43·39 |

*Statistics*

| | Mean | ρ | Standard deviation | Median | ρ' | Mode |
|---|---|---|---|---|---|---|
| Raw | 50·97 | 10·50 | 13·92 | 44·36 | 7·91 | 43·24 |
| Outlier rejected | 43·41 | 0·41 | 0·54 | 43·24 | 0·35 | 43·24 |

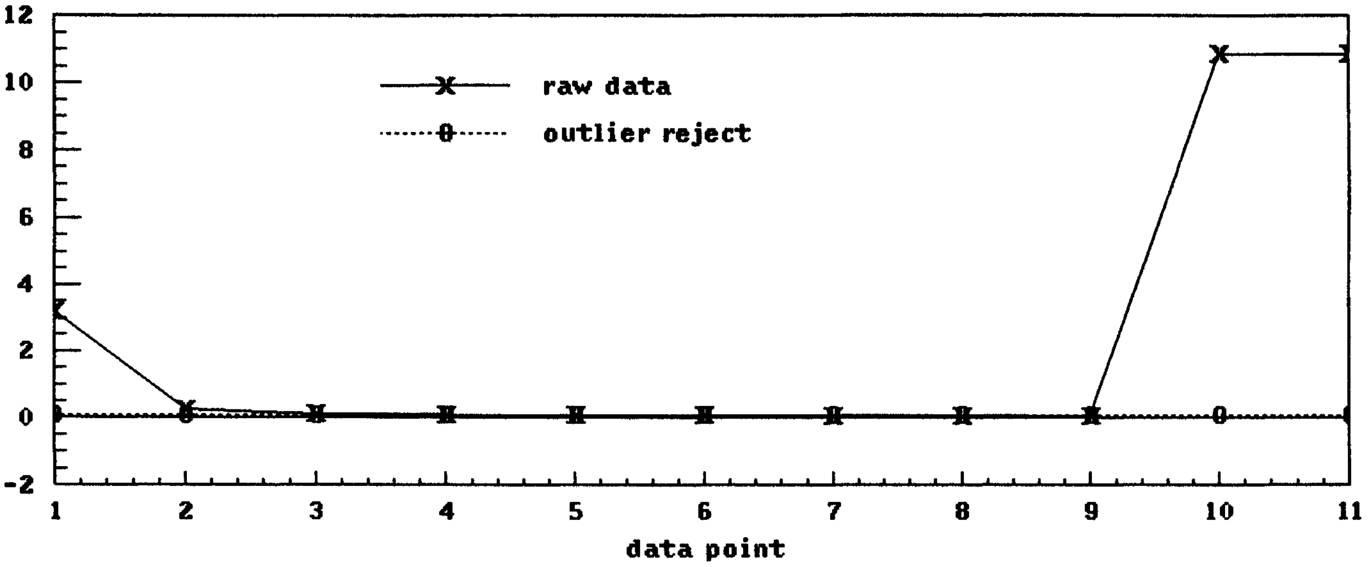

*Figure 1. Data set 1.*

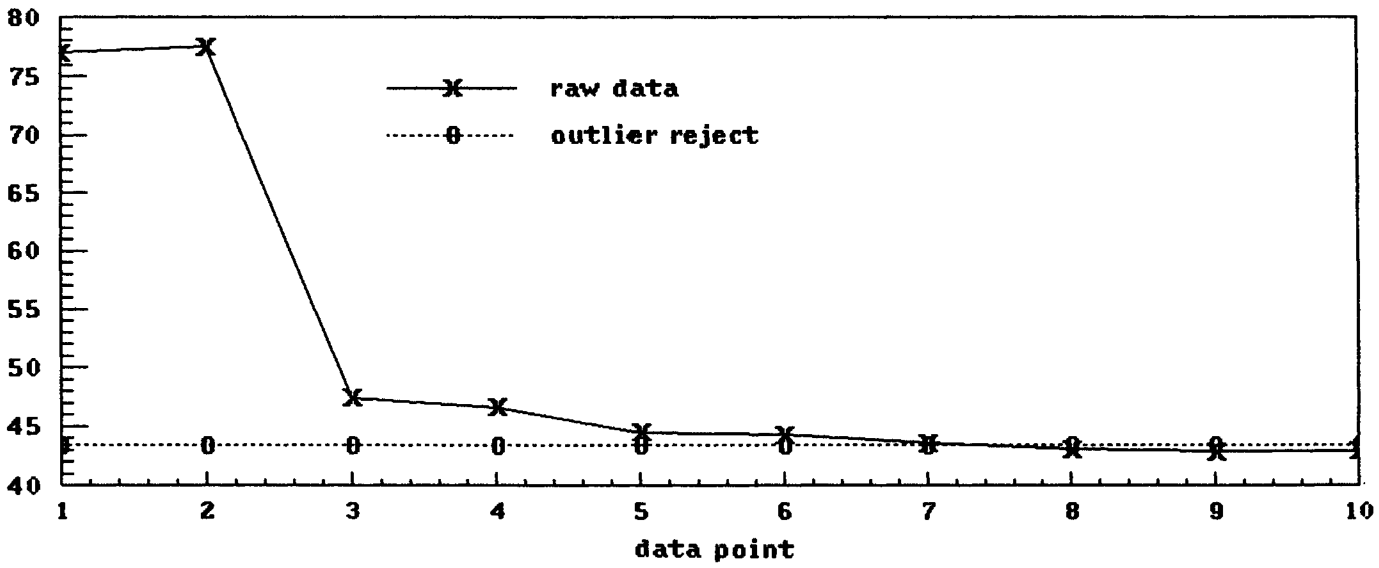

*Figure 2. Data set 2.*

rejection scheme presented above solves this by bounding.

The method of removing the linear trend in SMOOFT has hidden dangers. The straight line is constructed through the first and last data points. In the examples below, the first point is always from the previous experiment while the last may be from the following one. When restoring the linear trend after the frequency domain calculations, these points reintroduce unwanted characteristics.

### Examples

Two sets of experimental data are presented to illustrate the performance of the outlier rejection algorithm. The aim is to find the true or steady-state reading. The results are self-evident in the plots (see figures 1 and 2).

### Conclusions

A simple but effective outlier rejection routine has been presented. The routine is based on bounding data points to within the mean absolute deviation from the mode. This statistic does not seem to have been reported previously.

### References

1. Press W. H. *et al.*, *Numerical Recipes – The Art of Scientific Computing* (Cambridge University Press, 1989).
2. *PC-Matlab for MS-DOS Personal Computers* (The MathWorks Inc, South Natick, MA, USA, 1989).

